# The Influence of Iron Content on the Porosity of AlSi9 Alloy Intended for Alfining Piston Ring Inserts

**DOI:** 10.3390/ma17215181

**Published:** 2024-10-24

**Authors:** Jarosław Piątkowski, Stanisław Roskosz, Wiktoria Sapota, Sebastian Stach

**Affiliations:** 1 Faculty of Materials Engineering, Silesian University of Technology, Krasińskiego 8 Street, 40-019 Katowice, Poland; jaroslaw.piatkowski@polsl.pl; 2 Faculty of Science and Technology, Institute of Biomedical Engineering, University of Silesia in Katowice, Będzińska 39 Street, 41-205 Sosnowiec, Poland; wiktoria.sapota@us.edu.pl (W.S.); sebastian.stach@us.edu.pl (S.S.)

**Keywords:** Al–Si casting alloys, iron phases, porosity, automotive industry, pistons

## Abstract

Due to its tendency to increase the power of engines, improving their reliability and operational efficiency, the compression ring in combustion engine pistons is embedded in a cast iron insert, which is subjected to the process of “alfining”. This involves covering the insert with an Al–Si alloy, which increases the iron content. Research has shown that the β-Al_5_FeSi phases crystallizing in the area of the insert–piston connection are the main cause of an unstable connection between the silumin casting of the piston and the ring insert. Their unfavourable lamellar morphology and large dimensions are the main causes of weakening in the connection between the insert and the piston, resulting in an unacceptable number of defective products. It has also been found that up to approx. 0.59 wt.% Fe, the pore volume fraction is very small (up to 3%), and there is no correlation. However, after exceeding this value, both the volume fraction of the β-Al_5_FeSi phase and the number of pores increase monotonically to values of approximately 18% and 14%, respectively, and the correlation between the examined features is statistically significant. These results were compared with known theories of the influence of iron on the porosity of Al–Si alloys, showing that the precipitates of the β-Al_5_FeSi phase are more important in the porosity fraction than the two-layer oxide films called “bifilms”. This research was carried out and verified under industrial conditions in one of the largest piston foundries (Federal-Mogul Gorzyce sp. z o.o., F-MG) on a separate line intended for alfining ring inserts intended for combustion pistons.

## 1. Introduction

Iron is the most common and harmful impurity found in aluminium casting alloys. Its impact on mechanical properties, especially plastic properties, is particularly negative when its content exceeds approximately 0.4 wt.% (during gravity casting) [1,2,3,4,5] and approx. 1.8 wt.% (during die casting) [6,7,8,9,10]. This is because iron has low solubility in aluminium solid solutions (from 0.052% at 655 °C to 0.0052% at 450 °C) [11]. For this reason, it has a high tendency to combine with other elements, creating intermetallic phases of different stoichiometries, morphologies and sizes [11,12,13,14]. β-Al_5_FeSi (β-Fe) and α_H_-Al_8_Fe_2_Si phases are primarily undesirable. In Al–Si alloys with 0.8 to 1.2 wt.% Mg, in addition to the Mg_2_Si phase, there is a high probability of the crystallization of the Al_9_Fe_2_Si phase with a monoclinic structure and of the Al_8_FeMg_3_Si_2_ phase (ρ phase) with a hexagonal structure. In addition, metastable three-component phases of β-Al_4_FeSi (25.4 wt.% Fe and 25.5 wt.% Si) and β-Al_3_FeSi (33.9 wt.% Fe and 16.9 wt.% Si) crystallize, formed in conditions of thermodynamic imbalance [11]. In Al–Si–Cu and Al–Si–Cu–Mg alloys, in addition to Al_2_Cu, the Al_2_CuMg and Al_6_CuMg_4_ phases crystallize, existing in equilibrium with the solid-solution α(Al) and the Al_6_FeCu and Al_7_FeCu_2_ phases [15,16,17]. If the addition of nickel appears in Al–Si–Cu–Mg alloys, three-component phases, among others, Al_3_CuNi, Al_7_FeCu_2,_ Al_7_Cu_4_Ni, Al_9_FeNi and Al_3_(CuNi)_2_, and multi-component phases from the Al–Si–Cu–Mg–Ni–Fe system can crystallize apart from the Al_3_Ni phase [11,18].

Regardless of the chemical composition of aluminium alloys and iron content, the β-Al_5_FeSi phase is morphologically the worst. Due to its low coherence with the matrix and large dimensions, it increases the brittleness of the alloy, makes the machining of castings more difficult and reduces tensile strength [19,20,21,22]. In addition, the lamellar morphology of the β-Fe phase (acicular on the surface of the metallographic specimen) impairs the castability, ductility and corrosion resistance of aluminium alloys.

Another important reason for the reduced mechanical properties of aluminium alloy castings is porosity. According to research [23], an increase in porosity by approximately 1% reduces the R_m_ strength by approximately 40%. The formation of porosity in aluminium and its alloys is caused by a combination of two mechanisms [24,25,26,27]:

- Metal shrinkage during casting cooling, which causes a negative pressure of the liquid phase in the zone of dendrite growth of the solid-solution α(Al)—Darcy’s law [28];
- The dissolution and segregation of gases (oxides, sulphides, nitrides) in the metallic bath, which occur with different intensities, the most important being hydrogen [29,30,31].

If the gas concentration reaches the solubility limit, which decreases with pressure and temperature, then micropores nucleate. Their nucleation (homogeneous and heterogeneous) processes are known and described in the literature [26,29,32]. Findings show that the nucleation of pores is mainly caused by the contact of the liquid alloy with the mould walls, inclusions and the presence of gas bubbles. The chemical composition of the alloy is also important. Research [33] shows that elements such as copper, silicon and zinc reduce porosity. Chromium, cobalt, manganese and molybdenum have no significant effect on porosity, whereas magnesium, lithium and titanium cause a slight increase in porosity.

The factors of the (gravity or die) casting process also have an impact on the solubility of hydrogen and therefore on the porosity of castings. The most important ones include the following:

- Superheating temperature of the liquid alloy above the temperature T_liq._;
- Speed of the crystallization front;
- Temperature gradient;
- Range of the crystallization difference between temperatures T_liq._ and T_sol._ and crystallization time;
- Processes of refining the dendrites of the solid solution of α(Al) with titanium and boron mordants introduced separately or together;
- Processes for the modification of silicon crystals in a two-component eutectic system α(Al) + β(Si) (e.g., with strontium).

The influence of the above factors on porosity is also known and widely described in the literature, e.g., [25,26,27,29,32,33,34,35,36,37,38,39].

The establishment of a relationship between porosity and the presence of morphologically unfavourable iron phases in the alloy, including the β-Al_5_FeSi phase, raises greater concerns. Generally, there are two groups of theories:

- Porosity formation is caused by the presence of brittle, “plate-like” precipitates of the β-Al_5_FeSi phase in Al–Si alloys, mainly with an Fe content of over 0.4 wt.%. These precipitates, especially when they crystallize initially, block the interdendritic flow channels of the liquid alloy, and with a high iron content, they facilitate the nucleation of eutectic silicon crystals. This causes the deterioration of interdendritic permeability, especially that of secondary dendrites, and, consequently, an increase in the porosity of the castings [5,36,40,41,42,43];
- The formation of thin, two-layer oxide films, which exhibit a spherical or flattened shape during casting. This is due to the turbulent flow of the alloy, causing them to become tangled and folded. Campbell called them “bifilms” [19,20,44,45,46,47]. These oxides, due to the high sintering temperature (approx. 1900 °C) and the lack of wettability, especially on wavy, rough surfaces, are disconnected from each other, constituting a kind of “empty space” in the liquid. Once the alloy solidifies, these air gaps constitute crack and pore concentrators. The exact way in which these “bifilms” are formed is described in paper [48] using the example of the A356 alloy (Al-7Si-0.3Mg).

These views are often theoretical and have no direct experimental evidence. Moreover, in order to observe the phenomena of the heterogeneous nucleation of phase components in the areas of growth of secondary arms of aluminium dendrites or the processes of formation of thin oxide films and to characterize their surface, non-destructive 3D X-ray scanning methods are required. The obtained images must have a very high resolution (below 1 μm) to fully distinguish, for example, the morphology of bifilms. In addition, techniques for remelting and freezing the alloy need to be developed to capture the microstructure representing a given state of morphology of the phase components of aluminium alloys.

Therefore, it seems fully justified to undertake further research that would explain the phenomena causing porosity in Al–Si alloy castings and increase knowledge about the possible impact of iron phases on the porosity type.

The presented research is even more useful in the casting of aluminium alloys because of the following:

- It is based on the Al–Si alloy, which is used in real production processes, i.e., the “alfining” of cast iron ring inserts in engine pistons;
- All tests were verified in conditions of continuous industrial production;
- The obtained results can be used as guidelines for obtaining the correct connection of other bimetallic castings such as iron–aluminium alloys;
- It increases knowledge about the growing percentage of aluminium alloy recycling.

### 1.1. Piston Ring Inserts

Depending on the type of engine and operating conditions, there are different types of internal combustion engine pistons [49,50,51] made of different materials [52,53,54] and according to different technologies [55,56,57,58]. Most often, these are Al–Si foundry alloys with the addition of copper, magnesium, nickel and micro-additives, including manganese, chromium and titanium [59,60]. Due to their good strength and technological properties, these alloys are used, among others, for piston casting operating in an environment of high thermal and mechanical loads.

In the so-called ring zone of the piston, there are from one to four rings in two-stroke engines and from two to four in four-stroke engines. Their number depends, among others, on the compression ratio and engine design. These rings are designed to seal the combustion chamber and scrape excess oil from the cylinder walls. For this reason, they are often made of grey ductile or malleable cast iron [61,62].

**Figure 1:**
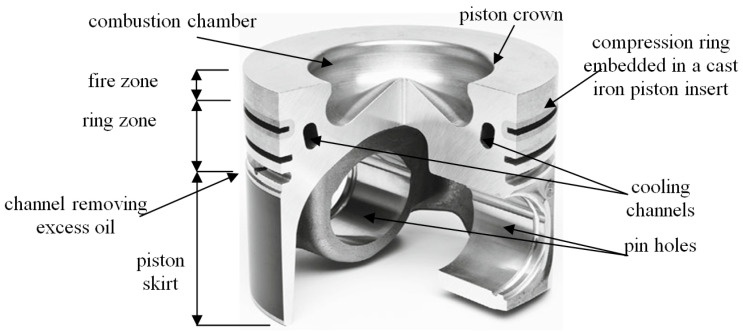
Main parts of the piston [63].

Due to the strengthening of the operating area of the piston ring zone and better heat dissipation, the (compression) ring is embedded in a cast iron insert (ring carrier). Until recently, pistons with a cast iron insert were used only in heavily loaded diesel engines, operating at a combustion pressure of 2.5 to 3.5 MPa, and in gasoline engines from 1.0 to 1.5 MPa [63]. Currently, due to increased engine power, increasing reliability during operation and meeting environmental emission standards, cast iron piston inserts (hereafter referred to as inserts) are used in all types of combustion engines. Ring inserts fulfil the following tasks (Figure 1):

- Reduce the temperature of the piston fire zone;
- Increase the tribological resistance of the upper ring zone of the piston;
- Improve the service life of piston rings, especially the compression ring;
- Increase the tightness between the compression ring and the cylinder liner;
- Prevent the compression ring from chipping;
- Improve combustion efficiency in the working chamber of the combustion engine.

In order for the ring insert to adhere better to the piston, it is necessary to create a tight connection between the insert and the Al–Si piston alloy. Therefore, the inserts are subjected to the so-called “alfining”. This is a process that involves briefly immersing the insert in an alloy to create a diffusion layer [63,64,65]. An AlSi9 alloy was used for alfining the inserts. After cooling, the alfined inserts are placed in a die-casting machine, filled with a piston alloy and then subjected to machining and further stages of piston production.

### 1.2. Problem Statement

**Figure 2:**
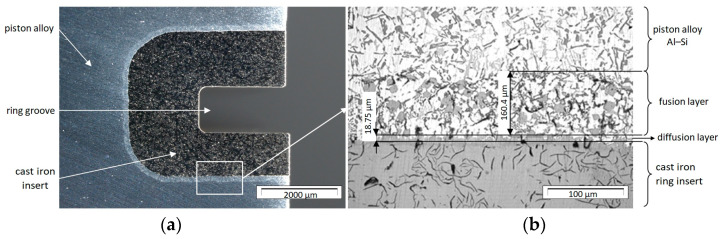
Connection of the insert with the piston: (**a**) macrostructure; (**b**) microstructure with a connection (fusion and diffusion) layer [63].

Statistical data from the final quality control of F-MG indicate that the most important causes of piston failures are casting defects. These defects are detected at the end of the production process during the final quality control of the pistons, which generates energy and time losses and reduces the efficiency of the production process. As research has shown, these defects result from the improper connection of the insert with the piston within the connecting layer (fusion and diffusion layers—Figure 2). Therefore, the control of the insert alfining process is a critical parameter to ensure the functionality of the piston and the correct operation of the piston–ring–cylinder system. The detection of defective insert–piston connections as soon as they occur makes it possible to reduce production costs in further stages of the piston manufacturing process. In addition, the load on machines and the consumption of materials in subsequent stages of production (mechanical and surface treatment of pistons) can be reduced.

## 2. Aim and Scope of the Research

The aim of this study was to demonstrate the cause-and-effect relationship between the iron content and porosity in the area of the layer connecting cast iron with the Al–Si alloy. A practical example of this is the combination of a cast iron ring insert with the AlSi11Cu4Ni3Mg piston alloy. Therefore, this research was carried out to identify the most important defects in the insert–piston connection and then to identify the causes of their occurrence.

This research also had a utilitarian value, which was to reduce the consumption of the AlSi9 alloy, i.e., “extend” its service life and reduce the number of defective products due to the unacceptable quality of the cast iron–silumin connection, as an example of bimetallic castings: cast iron–aluminium alloy.

To prove the adopted goals, the research was carried out using the deductive method, starting from carrying out industrial experiments, identifying the main defects in the area of the insert–piston connection and indicating the most important causes of these defects.

To identify the defects in the insert–piston connection, the production of pistons for a 1400 cm^3^ gasoline engine was chosen (the largest percentage of orders). The analysis of defects was carried out on a separate line for alfining inserts at F-MG.

The scope of research included:

- Assessing the percentage of casting defects in the insert–piston connection;
- Determining the causes of defects;
- Examining the microstructure of the insert–piston connection;
- Summarizing and drawing conclusions.

## 3. Materials and Research Methods

### 3.1. The Alfining Piston Ring Inserts

**Figure 3:**
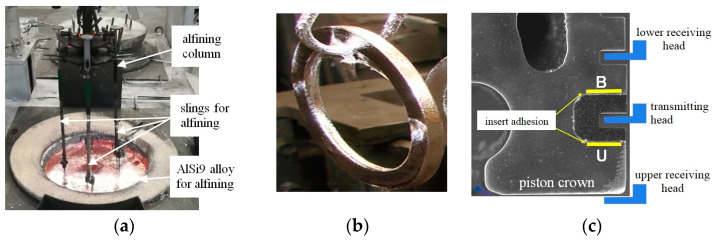
Alfining of ring inserts: (**a**) alfining station; (**b**) insert after alfining; (**c**) arrangement of measuring heads (property of Federal-Mogul Gorzyce sp. z o.o.).

The process of alfining the inserts was carried out on a semi-automatic line (Figure 3) under industrial conditions. The objects of the research were casting defects occurring on the contact surface of the insert with the piston. They were identified using the US Harrandt device and ultrasonic penetration research methods applied in F-MG.

In accordance with the TS1E-010-020 standard, the connection between the insert made of EN-GJLA-XNiCuCr15-6-2 cast iron (Table 1) and the piston cast from the AlSi11Cu4Ni3Mg alloy (Table 2) on its circumference is considered inappropriate if one of the following conditions is met:

- Tearing of the upper (U) surface of the insert;
- Tearing of the bottom (B) surface of the insert.

All of products were subjected to quality control tests of the insert–piston connection. The adhesion of the insert to the piston was tested on its upper (U) and lower (B) surfaces.

### 3.2. Microstructure Studies

Metallographic samples were cut from the upper and lower surfaces of the insert (Figure 3c). The samples were prepared based on the recommendations of the Buehler expert system. Metallographic observations were carried out on unetched samples using a MeF-2 Olympus GX-71 light microscope.

X-ray microanalysis was performed on a Hitachi S-4200 scanning microscope coupled with an EDS Voyager Noran X-ray spectrometer and on a Hitachi S-3400N scanning electron microscope equipped with an SE and BSE secondary electron detector. X-ray examinations were performed on a Philips X’Pert diffractometer using a lamp with a copper anode (λCu_Kα_-1.54178 Å) powered by a current of 30 mA at a voltage of 40 kV. The registration was performed using the “step-scanning” method with a step of 0.04° and a counting time of 10 s in the *2θ* angle range from 20° to 140°. The aperture of the incident beam was 1° and that of the diffracted beam 2°. Soller slots of 0.04 mm were used. Qualitative phase analysis was performed using the ICDD file. The DBWS 9807A program was used for the purposes of the analysis, and the Pearson VII function was used to describe the profile of diffraction lines.

The quantitative assessment of the microstructure was performed by quantitative metallography and image analysis using the ImageJ program [66,67,68]. For each sample, 10 baseline images were recorded over random fields of view. Binarization was performed using the Otsu method.

## 4. Results

### 4.1. Results of Chemical Composition

Based on the preliminary observation of the surface of the samples on which the chemical composition studies were carried out (Table 1, Table 2 and Table 3), EDS analysis and XRD, it was found that the structure of the samples was homogeneous on the surface. The results of testing the chemical composition of the cast iron from which the inserts were cast are presented in Table 1, those of the AlSi11Cu4Ni3Mg piston alloy in Table 2, and those of the AlSi9 alloy at the beginning of the alfining process in Table 3.

### 4.2. Results of Iron Content in the AlSi9 Alloy

The Pareto–Lorenz analysis shows that the high percentage of iron phases (42%) and porosity in this area (37%) are responsible for almost 80% of all defects at the insert–piston interface. The other defects constitute a total of approximately 20%. Therefore, further research was carried out to achieve the following:

- Identify iron phases and assess their impact on the quality of the connection: cast iron insert–Al–Si alloy;
- Assess the impact of porosity on the quality of the connection;
- Demonstrate a cause-and-effect relationship between the iron content and the porosity in the layer connecting the insert with the piston made of the AlSi11Cu4Ni3Mg alloy.

**Figure 4:**
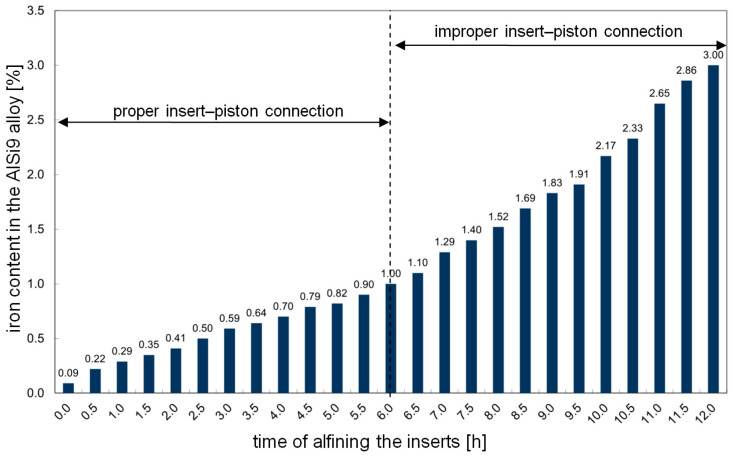
Change in the iron content of the AlSi9 alloy during the alfining of ring inserts.

Every hour, a sample of the chemical composition was taken from the AlSi9 alloy (Figure 4), the alfined inserts were placed in a die-casting machine and a trial batch (50 pieces) of pistons was cast. The batch of pistons cast every hour was subjected to metallographic examination.

After about 12 h of alfining the inserts, the measurements were stopped because the pistons cast in this period had a higher-than-expected percentage of defective products (in the area of the insert–piston connection). Therefore, it was necessary to replace the alloy for alfining with a new one.

Pistons cast in this way were conventionally divided into four groups:

- Group 1—pistons with an insert alfined using the AlSi9 alloy before 3 h. The percentage of defective products in this group is minimal.
- Group 2—pistons with an insert alfined using the AlSi9 alloy from 3.1 to 6 h. The percentage of defective products in this group is acceptable, but corrective actions in the piston production process are required.
- Group 3—pistons with an insert alfined using the AlSi9 alloy from 6.1 to 9 h. The percentage of defective products is unacceptable.
- Group 4—pistons with an insert alfined using the AlSi9 alloy from 9.1 to 12 h. The percentage of defective products is unacceptable due to abnormally high rejection.

### 4.3. Results of the Metallographic Investigation

**Figure 5:**
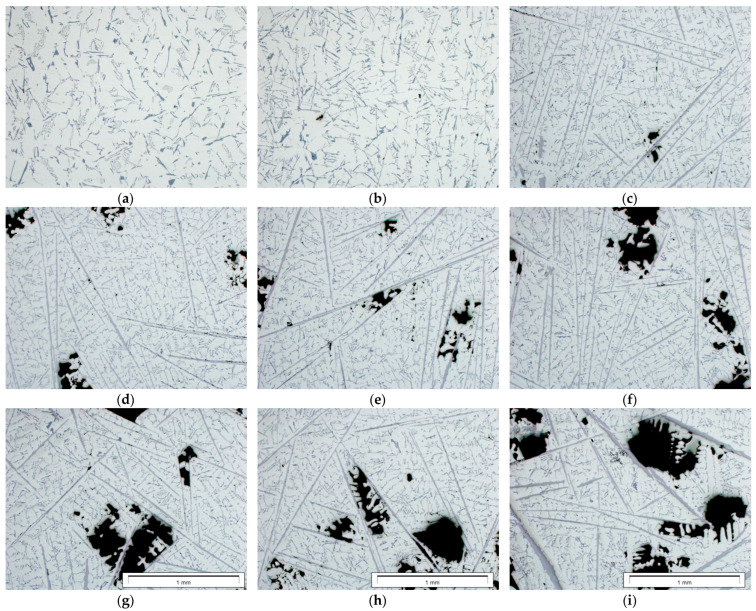
Images of microstructures of the AlSi9 alloy with the following iron contents: (**a**) 0.2, (**b**) 0.4, (**c**) 0.5, (**d**) 0.6, (**e**) 0.7, (**f**) 0.8, (**g**) 0.9, (**h**) 1.0 and (**i**) 1.2 (wt.%).

Examples of images showing the microstructures of the AlSi9 alloy with different iron contents are presented in Figure 5. The dark areas on the images are shrinkage pores.

**Figure 6:**
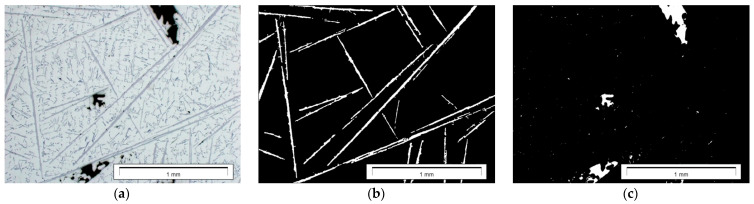
Microstructure of the AlSi9 alloy: (**a**) output image; (**b**) binary image of the β-Al_5_FeSi phase; (**c**) binary image of pores.

Binary images of the β-Al_5_FeSi phase were subjected to morphological transformations in order to correctly reproduce their size and shape (Figure 6). The measurement was made using the surface method. The results of measurements of the surface fraction of the β-Al_5_FeSi phase and pores were subjected to correlation analysis.

**Figure 7:**
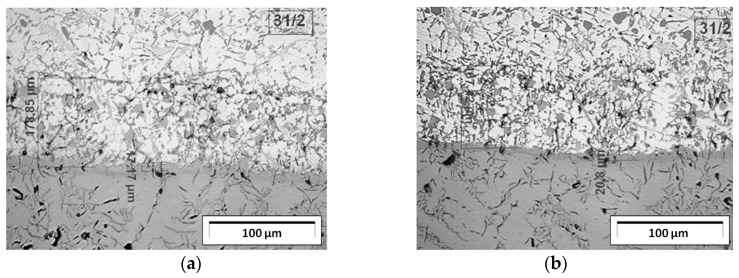

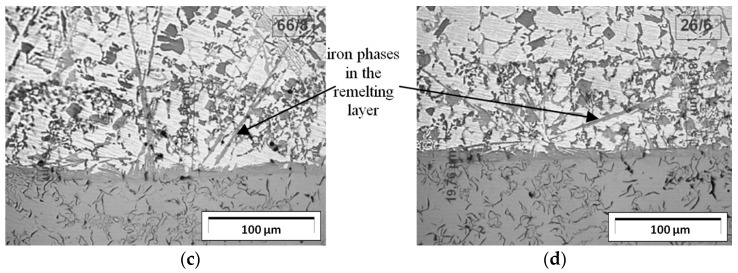
Microstructure of the insert–piston connection for the following times of using the AlSi9 alloy: (**a**) from 0 to 3 h (up to approx. 0.59 wt.% Fe); (**b**) from 3 to 6 h (from 0.6 to approx. 1.0 wt.% Fe); (**c**) from 6 to 9 h (from 1.01 to 1.84 wt.% Fe); (**d**) from 9 to 12 h (from 1.85 to 3.0 wt.% Fe).

To determine the causes of the lack of adhesion of the insert to the piston, microscopic examinations of the connection of the upper and lower surfaces of the insert with the piston were performed (Figure 3c). The microstructures of this connection representing each of the four groups are shown in Figure 7. This figure shows the following:

- For the first group of pistons (up to approx. 0.59 wt.% Fe in the AlSi9 alloy), few precipitates of iron phases were found in the area where the insert is connected to the piston. However, these phases did not negatively affect the connection.
- For the second group of pistons (from 0.6 to 1.0 wt.% Fe in the AlSi9 alloy), precipitates of iron phases were found in the area where the insert is connected to the piston. Their length ranged from approx. 100 to 400 μm.
- For the third group of pistons (from 1.01 to 1.84 wt.% Fe in the AlSi9 alloy), numerous long (from approx. 400 to 650 μm) and thick (from approx. 8 to 10 μm) precipitates of iron phases were found in the area where the insert is connected to the piston. A few porosity clusters were also found.
- For the fourth group of pistons (over 1.85 wt.% Fe in the AlSi9 alloy), numerous long (over 650 μm) and thick (from approx. 10 to 14 μm) precipitates of iron phases were found in the area where the insert is connected to the piston iron. Numerous porosity clusters were also found.

**Figure 8:**
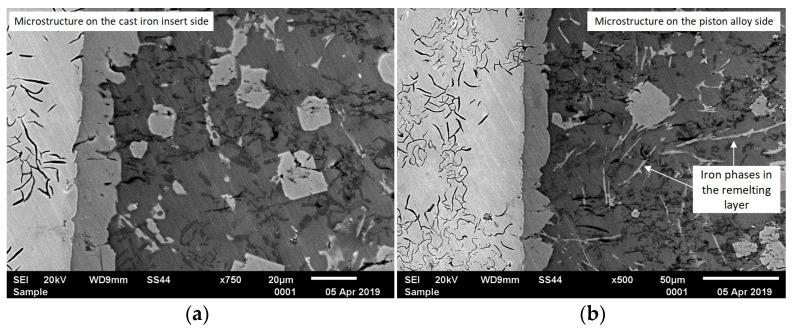
SEM microstructure of the insert–piston connection with the diffusion layer marked for the alfining time: (**a**) up to 6 h (up to approx. 1.0 wt.% Fe); (**b**) over 6 h (over 1.0 wt.% Fe).

The analysis of metallographic tests (Figure 7) shows that after approximately 3 h of using the AlSi9 alloy (0.6 wt.% Fe), single precipitates of iron phases begin to appear. However, their number in the connecting layer is so small that they do not weaken the insert–piston connection. The participation of these phases becomes problematic when the content of approximately 1.0 wt.% Fe is exceeded (6 h of using the AlSi9 alloy). Then, the iron phases are an important cause of poor insert–piston connection, resulting in an unacceptable percentage of defective products. To better illustrate the increased participation of iron phases, SEM tests of the insert–piston connection up to and above 1.0 wt.% Fe were performed, and the results are shown in Figure 8.

**Figure 9:**
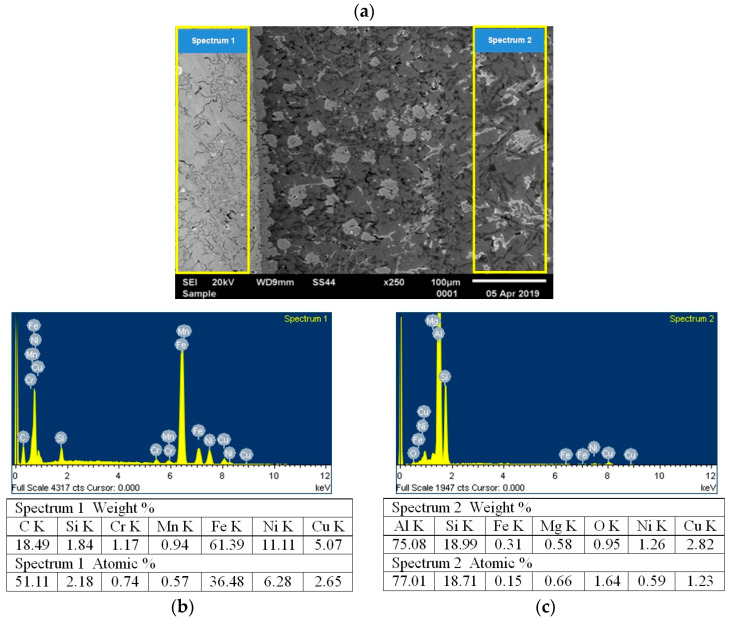
Results of testing the chemical composition of the layer connecting the insert with the piston: (**a**) connection microstructure; (**b**) micro-area and chemical composition on the insert side (cast iron); (**c**) micro-area and chemical composition on the piston alloy side (AlSi11Cu4Ni3Mg).

**Figure 10:**
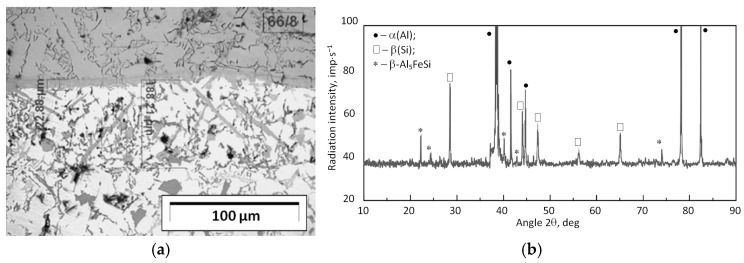
(**a**) Microstructure of the insert–piston connection; (**b**) XRD pattern.

In order to identify iron phases, the chemical composition of the layer connecting the insert with the piston was tested (Figure 9), and XRD tests were performed in this area (Figure 10).

XRD results show that in the remelting layer between the cast iron ring insert and the piston alloy, in addition to the solid-solution α(Al) and silicon crystals, which are part of the α(Al) + β(Si) eutectic system, there is also the β-Al_5_FeSi phase. This phase crystallizes in a lamellar form (acicular on the surface of the sample) with sharp edges and “pointed” corners, and its amount depends mainly on the iron content in the alloy.

**Figure 11:**
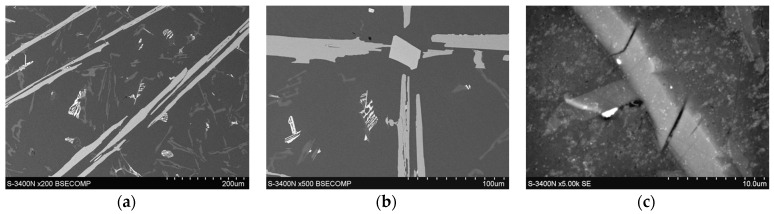
SEM microstructure of the remelting layer with visible cracks in the Al5FeSi phase: (**a**,**b**) at the interface; (**c**) transverse.

The examination of the AlSi9 alloy microstructure containing over 0.59 wt.% Fe also showed numerous cracks in the β-Al_5_FeSi phase precipitates. These cracks are located mainly at the interface between the Al_5_FeSi plate and the α(Al) matrix (Figure 11a,b) and throughout the Al_5_FeSi phase plate (Figure 11c).

### 4.4. Analysis of the Relationships Between the Iron Content, the Amount of the β-Al_5_FeSi Phase and Porosity in the AlSi9 Alloy

**Figure 12:**
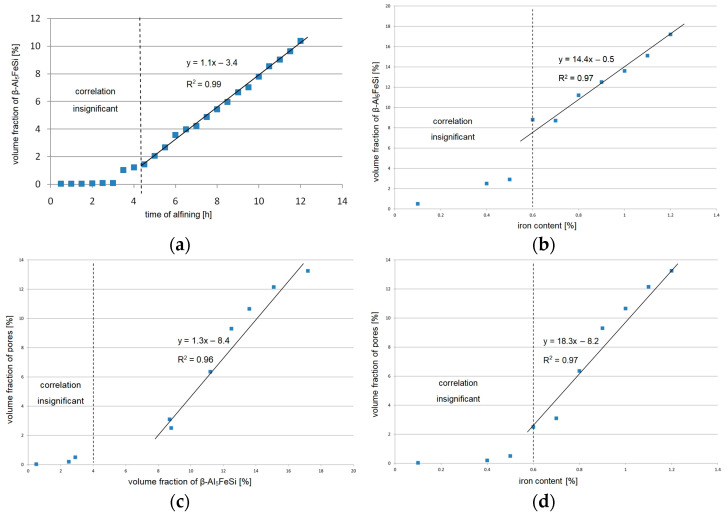
(**a**) The influence of the time of alfining on the amount of the β-Al_5_FeSi phase; (**b**) the influence of the iron content in the AlSi9 alloy on the amount of the β-Al_5_FeSi phase; (**c**) the influence of the amount of the β-Al_5_FeSi phase on porosity; (**d**) the influence of the iron content in the AlSi9 alloy on porosity.

Based on the measurements of the iron content, the amount of the β-Al_5_FeSi phase and the percentage of pores in the AlSi9 alloy in the layer connecting the insert with the piston, correlation charts were prepared, as shown in Figure 12.

Based on the data presented in Figure 12b, it was found that as the iron content in the AlSi9 alloy increases, the surface fraction of the β-Al_5_FeSi phase increases. With a low iron content (approx. to 0.59%), the amount of the β-Al_5_FeSi phase is small, and its volume fraction does not exceed 3%. However, when the Fe content ranges from 0.6 to 1.2 wt.%, the volume fraction of the phase increases from 8.2% to 17.8%. Such a large amount of the β-Al_5_FeSi phase significantly increases the shrinkage porosity in the area where the insert is connected to the piston. Figure 12c shows the influence of the volume fraction of the β-Al_5_FeSi phase on the volume fraction of pores. For a small amount of the β-Al_5_FeSi phase—up to 3%—porosity is low and ranges from 0.1 to 0.6%. However, when the surface fraction of the β-Al_5_FeSi phase ranges from 8.2% to 17.8%, porosity increases monotonically from the surface fraction of 2.8% up to 13.8%.

Figure 12d shows the effect of iron content on porosity. Up to approx. 0.59 wt.% Fe, the porosity in the alloy is low and does not show any correlation with the iron content. Above 0.6 wt.% Fe, the porosity in the alloy increases significantly, reaching a value of 17.8%.

Based on the conducted research, it was found that when the iron content exceeds 0.6%, large amounts of the β-Al_5_FeSi phase with lamellar morphology are released in the structure. This phase significantly increases the porosity of the castings, which is the main reason for the unstable connection between the silumin piston casting and the cast iron ring insert.

## 5. Summary

As the test results have shown, the alfining of piston ring inserts in the AlSi9 alloy causes a linear increase in the iron content (Figure 4). Using the alloy for up to approx. 6 h (1 wt.% Fe) does not cause any defects in the area of the insert–piston connection (due to the adopted criterion). After this time, as the iron content increases (caused by the diffusion of iron atoms into the AlSi9 alloy), the number of defective products also increases. An additional loss is the need to prematurely replace the AlSi9 alloy with a new one. The used alloy is treated as scrap and sold at a lower price compared to the feedstock, which generates financial losses. Thus, the alfining process of cast iron inserts is one of the main criteria determining the correct connection of cast iron ring inserts with piston castings made of Al–Si alloy. The percentage of casting defects on the contact surface of the insert with the piston indicates that the problem of proper connection between these elements is important from both process and technological points of view. Therefore, special attention should be paid to limiting and controlling the iron content in the AlSi9 alloy, especially after exceeding approximately 3 h of the alfining process. Research has shown that improper insert–piston connections are caused by two main reasons (accounting for almost 80% of all defects):

- An increase in the iron content in the AlSi9 alloy and therefore in the amount of the β-Al_5_FeSi phase;
- The accompanying porosity clusters, mainly of a shrinkage nature.

The dominant phase in the area where the insert is connected to the piston is the β-Al_5_FeSi phase. Its unfavourable lamellar morphology, dimensions and fragility weaken the connection between the insert and the piston, contributing to an increased percentage of defective products. An additional cause is cracks in the β-Al_5_FeSi (Figure 11).

Microscopic studies of the connection between the insert and the piston show that the β-Al_5_FeSi phases (at different iron contents) do not affect the grain size of the α(Al) solid solution in any way. No influence on the shape and size of the binary α(Al) + β(Si) eutectic was observed either.

As the iron content and the amount of the β-Al_5_FeSi phase increase, shrinkage porosity appears (Figure 5). The microstructures of the insert–piston connection as well as quantitative metallography tests and image analysis using the ImageJ program have shown that up to approximately 0.5 wt.% Fe, the amount of the β-Al_5_FeSi phase and porosity are low and do not exceed approximately 3% (Figure 12). After exceeding approximately 0.59 wt.% Fe, both the amount of the β-Al_5_FeSi phase and the porosity increase monotonically to approximately 18% for the β-Al_5_FeSi and approximately 14% for porosity. These results are slightly different from those presented in the paper [69]. Taylor et al. state that up to approx. 0.5 wt.% Fe, the porosity decreases and then increases. Unfortunately, the authors of this study do not explain the reasons for the obtained relationships.

Subsequent studies indicate that shrinkage pores are located in close proximity to the lamellar (acicular on the sample surface) precipitates of the β-Al_5_FeSi phase. With increasing content (over 0.59 wt.% Fe), individual pores combine into larger clusters that are “closed” around the β-Al_5_FeSi phases. It is clearly visible that the precipitates of the β-Al_5_FeSi phase hinder free access to fill interdendritic areas, causing high shrinkage porosity. Taking into account the results of microscopic examinations and thermal analysis [18], it can be concluded that up to approximately 0.3 wt.% Fe, the β-Al_5_FeSi phase is precipitated after the crystallization of the binary eutectic system α(Al) + β(Si) or is a component of the triple eutectic system α(Al) + Al_5_FeSi + β(Si). Its precipitates are short and do not make it difficult to fill the interdendritic spaces of α(Al). This is consistent with the results of research [69]. From approx. 0.3 to 0.59 wt.% Fe, the pre-eutectic crystallization of the β-Al_5_FeSi phase occurs, resulting in an increase in the length of its plates. Then, individual clusters of porosity appear, but they are not too large yet because dendrites of the solid-solution α(Al) have previously been released, effectively blocking (inhibiting) their growth. At over 0.6 wt.% Fe, the primary crystallization of the β-Al_5_FeSi phase probably occurs, the precipitates of which can grow freely, reaching a size of over 600 μm. In such a case, the primary β-Al5FeSi phases completely block the free flow of the liquid alloy, causing shrinkage and extensive porosity clusters with a morphology reflecting the dendrites of the α(Al) solid solution, which crystallize later. However, the precise determination of the temperature ranges of the variable crystallization sequence of the β-Al_5_FeSi phase (from the post-eutectic α(Al) + β(Si) to the primary one) requires additional thermal analysis (ATD) and calorimetric tests (DSC). However, it can be concluded that increasing the iron content in the AlSi9 alloy causes a change in the order of crystallization of the β-Al_5_FeSi phase and a related increase in the amount of shrinkage porosity.

Another issue is research carried out by Campbell indicating that the main sources of porosity are oxides formed when pouring the alloy from the furnace to a ladle or mould [19,20,44,45,46,47,48]. It should be noted, however, that the process of alfining the inserts is carried out in a preheated furnace without pouring the melt (Figure 3a). It is true that aluminium alloys are susceptible to the formation of oxides (liquid aluminium has a high affinity for oxygen), and oxidation occurs in milliseconds when the aluminium surface is in contact with atmospheric oxygen and moisture, but there is a compact surface on top of the liquid AlSi9 alloy mirror, preventing gases from entering the alloy. For this reason, the formation of “bifilms” in the AlSi9 alloy is unlikely. On this basis, it can be concluded that although the participation of oxide “bifilms” in the formation of porosity is possible, under these specific technological conditions, they do not have a significant impact on the formation of porosity.

## 6. Conclusions

The following conclusions have been formulated based on the research conducted:

1. Using the AlSi9 alloy for more than 6 h of alfining cast iron ring inserts causes an increase in the iron content (more than 1.0 wt.%), which crystallizes in the form of the β-Al_5_FeSi phase.
2. The β-Al_5_FeSi phase, with an unfavourable lamellar structure, is the cause of cracks and, above all, the reason for a significant increase in shrinkage porosity.
3. Up to approx. 0.59 wt.% Fe, the volume fraction of the β-Al_5_FeSi phase and the volume fraction of shrinkage pores have low values—up to approx. 3%.
4. A further increase in the iron content (more than 0.6 wt.%) increases the volume fraction of the β-Al_5_FeSi to 18% and the shrinkage porosity to 14%.
5. The main cause of poor insert–piston connection is the β-Al_5_FeSi phase and the accompanying shrinkage porosity. This microstructure of the layer connecting the insert with the piston results in a very high percentage of defective products.
6. The influence of the so-called oxide “bifilms” on porosity has not been confirmed.

## Figures and Tables

**Table 1:** Chemical composition of cast iron for piston ring inserts.

Cast Iron Designation	Element Content, wt.%									
	C	Si	Mn	Ni	Mg	Cr	P	S	Cu	Fe
GJLA-XNiCuCr15-6-2 (DIN EN 13835)	2.4–2.8	1.8–2.4	1.0–1.4	14.0–17.0	-	1.0–1.6	max. 0.08	max. 0.10	6.5–7.0	The rest

**Table 2:** Chemical composition of the AlSi11Cu4Ni3Mg piston alloy (rest–Al and other micro additives ^1^).

Element Content, wt.%													
Si	Cu	Mg	Ni	Fe	Mn	Zn	Ti	Pb	Zr	V	Sn	Cr	P ppm
10.0–11.5	3.5–4.3	0.5–1.0	2.2–3.2	Up to 0.5	Up to 0.2	Up to 0.1	Up to 0.05	Up to 0.05	Up to 0.05	Up to 0.03	Up to 0.05	Up to 0.03	60–100

^1^—up to 10.0 ppm Na; up to 10.0 ppm Ca; up to 10.0 ppm Sr; up to 150.0 ppm Sb.

**Table 3:** Chemical composition of the AlSi9 alloy for alfining ring inserts (rest–Al).

Element Content, wt.%							
Si	Mg	Cu	Mn	Fe	Ni	Zn	Others
8.5–10.00	up to 0.03	up to 0.20	up to 0.12	up to 0.15	up to 0.05	up to 0.05	up to 0.02

## Data Availability

The original contributions presented in the study are included in the article, further inquiries can be directed to the corresponding author.
